# Effector-triggered post-translational modifications and their role in suppression of plant immunity

**DOI:** 10.3389/fpls.2012.00160

**Published:** 2012-07-16

**Authors:** Andrew J. M. Howden, Edgar Huitema

**Affiliations:** Division of Plant Science, College of Life Sciences, University of Dundee at The James Hutton Institute, Dundee, UK

**Keywords:** effector, PAMP-triggered immunity, effector-triggered susceptibility, post-translational modifications, direct effector-triggered modification, indirect effector-triggered modification

## Abstract

Plant–pathogen interactions feature complex signaling exchanges between host and microbes that ultimately determine association outcomes. Plants deploy pattern recognition receptors to perceive pathogen-associated molecular patterns, mount pattern-triggered immunity (PTI), and fend off potential pathogens. In recent years an increasing number of defense-signaling components have been identified along with a mechanistic understanding of their regulation during immune responses. Post-translational modifications (PTMs) are now thought to play a crucial role in regulating defense signaling. In a bid to suppress PTI and infect their host, pathogens have evolved large repertoires of effectors that trigger susceptibility and allow colonization of host tissues. While great progress has been made in elucidating defense-signaling networks in plants and the activities of effectors in immune suppression, a critical gap exists in our understanding of effector mechanism-of-action. Given the importance of PTMs in the regulation of defense signaling, we will explore the question: how do effectors modify the post-translational status of host proteins and thus interfere with host processes required for immunity? We will consider how emerging proteomics-based experimental strategies may help us answer this important question and ultimately open the pathogens’ effector black box.

## INTRODUCTION

Within their natural environment, plants are continuously challenged by a diverse array of pathogens such as viruses, bacteria, fungi, and oomycetes as well as nematodes and insects. In most cases, infection or disease is limited upon the perception of pathogen- or microbe-associated molecular patterns (PAMPs or MAMPs) by pattern recognition receptors (PRRs; Boller and Felix, 2009). Recognition results in PAMP or pattern-triggered immunity (PTI) and features a marked shift in transcriptional activity toward defense, as well as the production and secretion of defense-associated proteins and metabolites leading to increased levels of resistance (Jones and Dangl, 2006; Zipfel, 2009). With some of the principal players and processes identified in plant immune signaling networks, only now we are starting to appreciate the critical roles of regulatory mechanisms that ensure an appropriate response to a given biotic stress.

Protein post-translational modifications (PTMs) are ubiquitous in cell signaling networks and enable rapid alterations to the protein complement of the cell without need for new protein synthesis. In addition, PTMs provide enormous diversity to the proteome, allowing cells to respond with flexibility to a stimulus. PTMs regulate a wide array of processes within plants including growth, development, flowering, and defense (Kwon et al., 2006). Phosphorylation, ubiquitination, and SUMOylation have emerged as pivotal PTMs that plants employ to target and control the activity of immune regulators (Stulemeijer and Joosten, 2008). These findings (reviewed elsewhere in this issue) have highlighted the intricacies of an immune system that has adapted to an environment in which plants are bombarded by commensal, symbiotic as well as pathogenic organisms.

In a select few cases, plants are successfully invaded and colonized by microbes, which in turn can lead to the manifestation of disease. Disease development ultimately results from the perturbation of immune signaling networks, suppression of defense responses and modulation of metabolism in the host (Jones and Dangl, 2006; Dodds and Rathjen, 2010). Pathogens have evolved strategies to actively evade or suppress immunity. Given the importance of PTMs in regulating immune signaling networks, it is likely that pathogens specifically target and perturb host PTM pathways implicated in defense.

**FIGURE 1:**
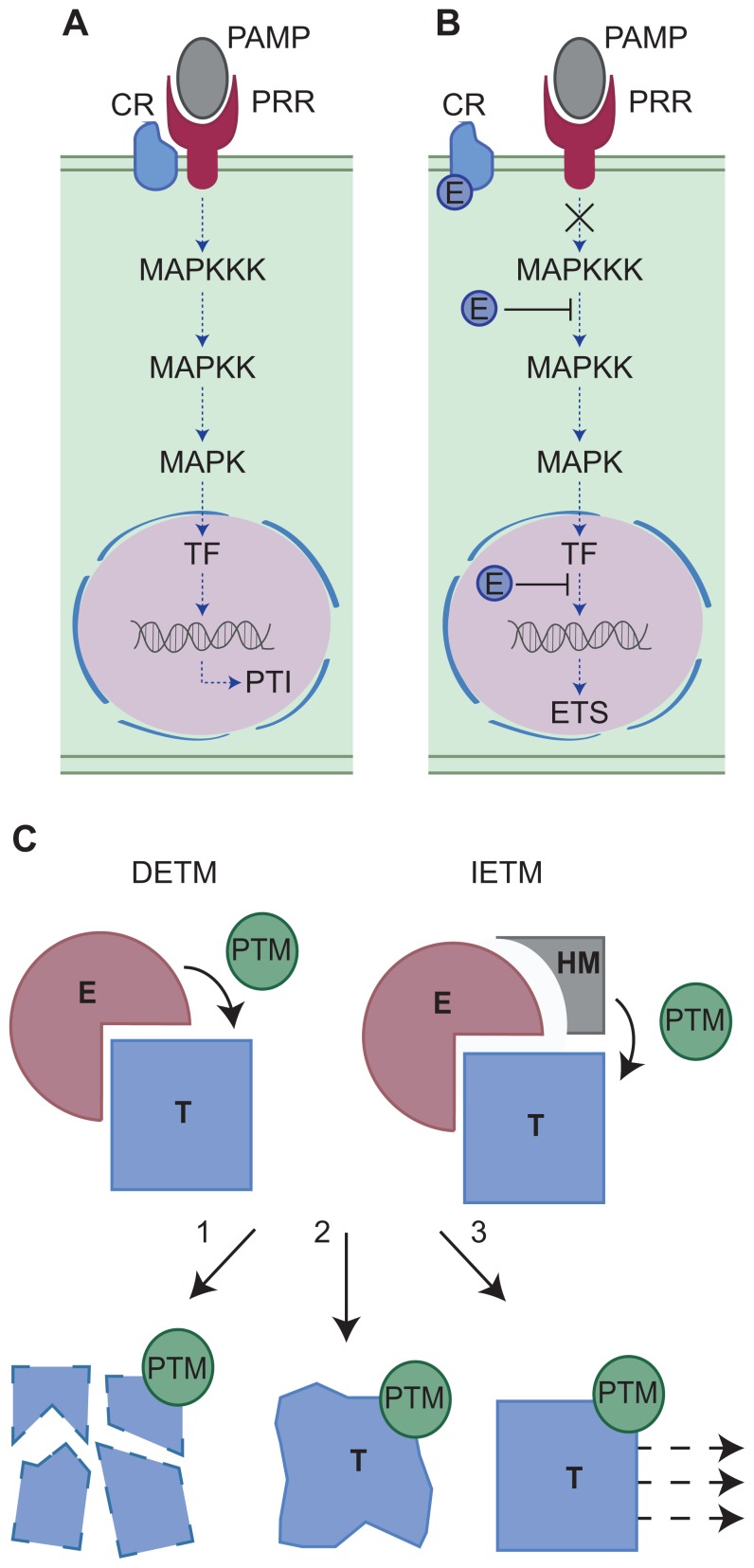
**(A,B)** Modulation of host defense-signaling networks by pathogen effectors. One example of effector mediated host defense modulation is the manipulation of mitogen-activated protein kinase (MAPK) phosphorylation cascades. **(A)** Host defenses may be activated by the perception and binding of pathogen-associated molecular patterns (PAMPs) by host pattern recognition receptors (PRRs) with the aid of a co-receptor (CR). Successful PAMP perception triggers a MAPK phosphorylation cascade resulting in transcription factor (TF) activation, defense gene expression and PAMP-triggered immunity (PTI). However, pathogen effectors (E) can manipulate this signaling pathway at a number of key steps **(B)** resulting in effector-triggered susceptibility (ETS). For example, the MAPK cascade can be blocked either by the effector perturbing CR-PRR activities or by the effector modifying MAPKs directly. Alternatively, nuclear targeting effectors may block defense gene induction, ultimately leading to enhanced susceptibility and the onset of disease. **(C)** Two models for effector-triggered post-translational modifications. In Model 1 (direct effector-triggered modification – DETM) the effector (E) binds the host target protein (T) and directly catalyzes its post-translational modification (PTM). In Model 2 (indirect effector-triggered modification – IETM) the effector binds the target protein and recruits a host machinery (HM) which catalyzes target PTM. The modified host protein may then be subject to proteasome mediated degradation (1), altered structural confirmation and activity (2), or re-localization (3).

The last decade has seen the identification of pathogen encoded secreted proteins (effectors) that upon secretion, manipulate host processes, perturb signaling, and induce effector-triggered susceptibility (ETS; Kamoun, 2007; Hann and Rathjen, 2010). During infection, pathogens secrete effectors that accumulate in the host intercellular space (apoplastic effectors) and target apoplastic or surface exposed host components. Pathogens can also secrete effectors that translocate across the host cell membrane (intracellular effectors) and upon delivery, travel to discrete subcellular compartments or organelles to target resident cellular processes (Kamoun, 2006; Block et al., 2008). Both effector classes are thought to modulate host (defense) signaling and perturb cellular processes required for PTI, ultimately leading to ETS. One example of effector driven host defense modulation is the manipulation of mitogen-activated protein kinase (MAPK) phosphorylation cascades, which ultimately leads to altered defense gene expression and enhanced susceptibility (**Figures 1A,B**).

In recent years great progress has been made in elucidating defense-signaling networks in plants and identifying those pathogen effectors that can suppress immunity. However, an understanding of the mechanism-of-action of most effectors remains elusive. Given the lack of models describing effector mode-of-action and the importance of PTMs in defense signaling, we will explore the question: how do effectors modify the post-translational status of host proteins and thus interfere with host processes required for immunity? In this context, we will aim to consider how emerging proteomics-based experimental strategies may help us answer this important question, and ultimately open the pathogens’ effector black box.

## TWO MODELS DESCRIBE EFFECTOR-TRIGGERED PTMs

Effector-triggered susceptibility is achieved through the interaction between a pathogen effector and its host target that eventually impinges on immune signaling. Mechanistically, this interaction results in a modification of the target and its cellular fate (Kamoun, 2007). The host target protein may be modified by the addition or removal of a chemical group (PTM). The resulting PTM may trigger target protein degradation, altered protein conformation and activity or re-localization (Kwon et al., 2006). Effector-triggered target modification requires an enzymatic activity that is either provided by the effector or by a given host cellular pathway. These observations infer the presence of at least two mechanistic models (**Figure 1C**) that help explain effector-triggered target modification. Both simplified models will be discussed and explored here and examples supporting both models are also provided (**Table 1**).

**Table 1:** Examples of direct effector-triggered modification (DETM) and indirect effector-triggered modification (IETM).

Effector	Pathogen/host	Target	Effector activity	Reference
**DETM**
AvrPtoB	*Pst/S.l.* and *A.t.*	FLS2, BAK1, FEN, CERK1	E3 ubiquitin ligase	Rosebrock et al. (2007), Goehre et al. (2008),Shan et al. (2008), Gimenez-Ibanez et al. (2009)
AvrPphB	*Pst/S.l.* and *A.t.*	PBS1	Cysteine protease	Zhang et al. (2010)
AvrRpt2	Pst/S.l. and A.t.	RIN4	Cysteine protease	Mackey et al. (2002, 20003,
AvrAC	*Xcc*/brassicas	BIK1, RIPK	Uridylyl transferase	Feng et al. (2012)
HopAI1	*Pst/S.l.* and *A.t.*	MPK3, MPK6	Phosphate lyase	Zhang et al. (2007)
HopU1	*Pst/S.l.* and *A.t.*	GRP7	Mono-ADP-ribosyltransferase	Jeong et al. (2011)
**IETM**
AvrB	*Pst/S.l.* and *A.t.*	RIN4	Recruits host protein kinase	Liu et al. (2011)
AvrRpm1	*Pst/S.l.* and *A.t.*	RIN4	Recruits host protein kinase	Liu et al. (2011)
HopM1	*Pst/S.l.* and *A.t.*	AtMIN7	Recruits host protein kinase	Nomura et al. (2006)
**Unknown**
Avr3a	*P.i./S.t.*	CMPG1	Stabilizes target	Bos et al. (2010)

*Pathogen and host names: Pst, Pseudomonas syringae pv. tomato DC3000; Xcc, Xanthomonas campestris pv. Campestris; P.i., Phytophthora infestans; S.l., Solanum lycopersicum, A.t., Arabidopsis thaliana; S.t., Solanum tuberosum.*

### MODEL 1: DIRECT EFFECTOR-TRIGGERED MODIFICATION

The first model (which we have named direct effector-triggered modification or DETM) assumes that a direct interaction between effector and target, combined with an enzymatic activity carried by the effector, ensures modification of the target. In recent years a number of plant pathogen effectors have been shown to modify host targets by direct interactions combined with a catalytic activity carried by the effector. The *Pseudomonas syringae* pv. *tomato* DC3000 (*Pst*) effector AvrPtoB features an N-terminal kinase binding motif that aids binding to host receptor like kinases FLS2, BAK1, FEN, and CERK1 (Rosebrock et al., 2007; Goehre et al., 2008; Shan et al., 2008; Gimenez-Ibanez et al., 2009). The AvrPtoB C-terminus has been shown to exhibit E3 ubiquitin ligase activity, and is responsible for the degradation of FEN during *Pst* infection of tomato (Rosebrock et al., 2007). AvrPtoB binding to the PRRs FLS2 and CERK1, and their co-receptor BAK1 also leads to their degradation and enhanced virulence of *Pst* on *Arabidopsis* (Goehre et al., 2008; Shan et al., 2008; Gimenez-Ibanez et al., 2009). AvrPtoB induced degradation of factors important for immunity leads to increased susceptibility to *Pst* on both tomato and *Arabidopsis* (Rosebrock et al., 2007; Goehre et al., 2008), providing a compelling illustration of effector catalyzed modification events that lead to ETS. Besides targeting proteins to the host proteasome for degradation, effectors can also directly eliminate targets from the host cell. AvrRpt2 encodes a cysteine protease that is delivered into host cells during *Pst* infection where it is activated by the eukaryotic host factor cyclophilin (Coaker et al., 2005). Activated AvrRpt2 associates with the host plasma membrane and releases RIN4 from the host cell membrane by proteolysis. RIN4 dissociation from the membrane results in enhanced susceptibility, provided that RPS2, one of two resistance proteins guarding RIN4 is absent (Mackey et al., 2002, 2003; Kim et al., 2005).

Besides proteolytic degradation, DETM can also alter host protein phosphorylation status. MAPKs link PAMP perception to downstream defense gene expression (Pitzschke et al., 2009). The *Pst* effector HopAI1 interacts with the *Arabidopsis* MAP kinases MPK3 and MPK6. During PTI, both MPK3 and MPK6 are activated by the phosphorylation of a threonine residue by upstream MAPKKs. HopAI1 phosphate lyase activity however, removes the phosphate group from these residues, preventing MPK3 and MPK6 activation by PAMP induced MAPKKs. Since phosphate group removal cannot be reversed, both MPK3 and MPK6 are effectively inhibited, leading to dampening of PTI activated MAPK signaling cascades (Zhang et al., 2007).

### MODEL 2: INDIRECT EFFECTOR-TRIGGERED MODIFICATION

Many C-terminal effector domains do not exhibit any sequence similarity to known enzymes. While some enzymatic functions have been elucidated after solving and comparing effector structures to known catalytic enzymes, there are an increasing number of effectors for which enzymatic function remains elusive. Although the presence of an unknown enzymatic function can never be excluded, this observation raises the possibility that in such cases, effectors modify their targets with the help of host-encoded enzymes, in a process that we have termed indirect effector-triggered modification (IETM). Examples of such mechanisms are sparse but one early report emanated from studies on the human papilloma virus oncoprotein E6. E6 was identified in oncogenic HPV strains and detailed studies of this protein led to the observation that in host cells, E6 recruits an E3 ubiquitin ligase that in turn, ubiquitinates the tumor suppressor protein p53. E6 induced p53 ubiquitination was found to mark the tumorigenesis suppressor for proteasomal degradation, providing a molecular explanation for the onset of cancer in infected cells (Scheffner et al., 1990).

One of the best characterized examples of IETM in plant–microbe interactions features the *P. syringae* effectors AvrB and AvrRpm1. Delivery of either AvrB or AvrRpm1 results in phosphorylation of its molecular target RIN4. Attempts aimed at demonstrating kinase activity of either effector have failed. However, the host kinase RIPK (RPM1-induced protein kinase) has been found to form a complex with RIN4 and AvrB, and this observation has helped to explain the effector dependent phosphorylation of RIN4. Delivery of AvrB inside host cells leads to recruitment of RIPK to an AvrB–RIN4–RIPK complex. RIPK phosphorylates RIN4 threonine residue 166 presumably suppressing PTI in the absence of RPM1 (Chung et al., 2011; Liu et al., 2011).

Another interesting example of IETM is the activity of the *Pst* effector HopM1. Within the host HopM1 interacts with the defense-associated protein AtMIN7, targeting it for degradation by the host proteasome and resulting in impaired cell wall-associated defenses. HopM1 is thought to act as an adapter protein which shows no similarity to proteins involved in ubiquitination or proteolysis but instead recruits the host machinery to selectively remove a key defense protein (Nomura et al., 2006). These findings combined with an immense but yet elusive repertoire of effector domains with unknown function, hint that IETM is a common mechanism that drives effector induced target modifications during host–microbe associations.

There is now increasing evidence supporting the DETM and IETM models for effector activity. However, in some cases despite the effector target having been identified, the modification events remain elusive. The *Phytophthora infestans* RXLR effector AVR3a interacts with the host E3 ubiquitin ligase CMPG1, an interaction that protects this target from degradation and leads to its stabilization in host cells. The presence of AVR3a induces an increase in CMPG1 molecular mass, suggesting that specific PTM events underpin stabilization. The presence of AVR3a suppresses INF1-induced cell death and PTI, suggesting that CMPG1 modification perturbs key PTI signaling steps (Bos et al., 2010). Despite these observations and the recent elucidation of structures for two members from the AVR3a protein family in *Phytophthora capsici* (Boutemy et al., 2011; Yaeno et al., 2011), the exact function of AVR3a remains to be determined. The observation that AVR3a cell death suppression activity can be uncoupled from R3a (a NBS-LRR) mediated recognition of AVR3a, may suggest the presence of additional host factors that are recruited by AVR3a and are guarded by R3a.

## EMERGING PROTEOMICS-BASED EXPERIMENTAL STRATEGIES WILL HELP US EXAMINE THE ROLE OF PTMs IN EFFECTOR ACTIVITY

With the availability of an ever increasing array of pathogen and host genomes, great advances have been made in the identification of putative pathogen effectors and the disease signaling networks these proteins may impact upon. Considering the immense diversity in functional effector domains, we are only now beginning to appreciate the vast but yet unexplored repertoire of novel activities encoded by microbial effectors and their possible roles in ETS. Given the recent advances in proteomics approaches, we will discuss experimental approaches that can be employed to further understand effector function in the context of the models described here.

## *IN SITU* DETECTION OF EFFECTORS AND THEIR PUTATIVE TARGETS

Effectors have been shown to target specific subcellular compartments where they modulate distinct host processes (Kamoun, 2006; Block et al., 2008). The identification of host compartments targeted by pathogen effectors thus forms a critical requirement to understand function. Confocal microscopy-based localization studies are a powerful means to deduce effector targeting in plant cells. It should be noted however that during localization experiments, conditions that reflect a given host–microbe interaction cannot be easily reconstituted. Monitoring the proteome of host organelles during the course of infection represents a powerful tool for studying effector-triggered host modifications *in situ*. Organelle enrichment strategies, combined with LC–MS/MS would enable the identification of effectors which localize to particular organelles while simultaneously monitoring the relative abundance and post-translational status of host proteins. Recently, Drakakaki et al. (2012), used vesicle affinity purification combined with mass spectrometry to identify proteins of the trans-Golgi network in *Arabidopsis*. This method provided the sensitivity to reveal novel protein complexes and trafficking components. Similar strategies may be employed for studying organelle proteomes during infection.

Subcellular fractionation has the added benefit of enriching the protein sample for fractions of interest, making the detection of low abundance signaling proteins and their PTMs more achievable. Such strategies will have to be combined with quantitative proteomics techniques in order to monitor the relative abundance of proteins at a given time or location. Quantitative proteomics is technically challenging in plants due to their autotrophic nature and the resulting difficulties associated with whole proteome labeling. However, strategies are now available for labeling plant cell cultures using either isotopically labeled nitrogen compounds (^15^N; Keinath et al., 2010) or stable isotope labeling by amino acids (SILAC; Schuetz et al., 2011), while whole plants may be isotopically labeled using ^15^N (Schaff et al., 2008). In addition, label free proteomics is also likely to become more routine with improvements in the accuracy and reproducibility of mass spectrometers (Oeljeklaus et al., 2009).

## ENRICHING FOR PTMs

 Our indirect model (IETM) of effector-induced PTMs raises a range of issues which should be considered when examining effector–target interactions. The indirect modification model assumes that the effector binds a host target protein and recruits host machinery which then catalyzes target PTM. Detecting the interaction between three partners (effector, target, and host machinery) is a challenge since the stoichiometry of each partner may not be equal. Conventional protein–protein interaction experiments using yeast-2-hybrid or tandem affinity purification may only detect the most abundant partners within this interaction. The target protein may be expressed at relatively low levels and the stoichiometry of the PTM often means that the modified protein represents a small proportion of the total pool for that protein. Enrichment strategies are therefore desirable if one wishes to survey the proteome for targets with a specific PTM. A range of strategies are now available for enriching proteins or peptides for specific PTMs, including antibody-based affinity enrichment and ionic interaction-based enrichment (Larsen et al., 2005; Zhao and Jensen, 2009). Nühse et al. (2007) used ionic interaction-based enrichment combined with a quantitative proteomics strategy to examine dynamic changes in the phosphorylation of *Arabidopsis* plasma membrane proteins treated with the bacterial elicitor flagellin. In a more recent study Mithoe et al. (2012) used ^15^N metabolic labeling in combination with immunity-affinity purification to enrich and quantify tyrosine phosphorylated peptides upon flagellin perception in *Arabidopsis*. Combining tandem affinity purification with PTM enrichment strategies may provide the sensitivity to detect subtle effector induced PTM events.

## OUTLOOK

The ability of pathogens to modify the post-translational state of host proteins represents a powerful means for the pathogen to tip the balance from immunity to susceptibility. Since most effectors have yet to be assigned a precise function, and given the enormous diversity that exists among those effectors identified to date, the expectation is that the future will see the identification of alternative effector-triggered PTMs and their substrates. However, a bottleneck is now forming in the characterization of effector activities, and is likely to build further as whole genome sequencing projects identify ever increasing numbers of putative effectors. The use of proteomics to monitor effector localization and host proteome dynamics is likely to emerge as a crucial tool that will enable effector activities to be linked with host PTM signaling pathways. An improved understanding of the mechanisms by which pathogens use their effector repertoire to manipulate host defense signaling, will prove invaluable for developing plant lines with improved pathogen resistance.

## Conflict of Interest Statement

The authors declare that the research was conducted in the absence of any commercial or financial relationships that could be construed as a potential conflict of interest.
